# Unilateral amelanotic conjunctival malignant melanoma: a case report

**DOI:** 10.1186/s13256-024-04729-3

**Published:** 2024-09-03

**Authors:** Marcelo Ayala, Kalliopi Erripi, Iva Johansson

**Affiliations:** 1 Eye Department, Skaraborg Hospital, Sahlgrenska Academy, Gothenburg University & Karolinska Institute, 541 85 Skövde, Sweden; 2 Eye Department, Sahlgrenska University Hospital, Gothenburg, Sweden; 3 Department of Clinical Pathology, Sahlgrenska University Hospital, Sahlgrenska Academy, University of Gothenburg, Gothenburg, Sweden

**Keywords:** Amelanotic, Malignant melanoma, Eyelids, Unilateral, Conjunctiva, Eye

## Abstract

**Introduction:**

Cutaneous malignant melanomas rarely occur in the eye, usually in the eyelids or the conjunctiva. Conjunctival malignant melanomas are even rarer. Most melanomas are dark in color as they are pigmented. However, amelanotic conjunctival malignant melanomas, a scarce variant of the cancer, can be challenging to diagnose accurately.

**Case presentation:**

We present two cases of white Caucasian Swedish-born women who were diagnosed with unilateral amelanotic malignant melanoma in the conjunctiva of the eye. In the first case, the patient was an 81-year-old woman who was suffering from redness and foreign body sensation in the left eye. The initial diagnosis was blepharitis. Three biopsies were taken, which showed malignant melanoma in the eyelid and the conjunctiva. Unfortunately, the eye and the rest of the orbit could not be saved, and the patient had to undergo an orbital exenteration. In the second case, the patient was a 50-year-old woman, and the tumor was localized in the temporal conjunctiva of the left eye. The initial diagnosis was pinguecula, but at the time of surgery, the physician suspected conjunctival intraepithelial neoplasia. The tumor was not completely removed, so adjuvant brachytherapy and local chemotherapy were used. The eye was preserved. No neck and/or lung metastasis was detected in either case at the time of diagnosis.

**Conclusions:**

Conjunctival amelanotic malignant melanomas should be suspected when tumors are present in the eye and/or the eyelids. By suspecting amelanotic malignant melanoma, the delay in treatment can be shortened. Treating them as soon as possible is essential to minimize the risk of metastasis.

## Background

Conjunctival malignant melanoma is a rare and aggressive type of cancer that originates in the pigmented cells of the conjunctiva. The overall incidence of conjunctival melanoma was 0.5/1,000,000/year, and it increased in Denmark over 52 years as the population got older [1]. In Sweden, a study published in 1992 reported an incidence rate of 0.024/100,000/year [2]. The amelanotic variant of conjunctival malignant melanoma is even rarer, accounting for around 10% of all conjunctival melanomas [1, 3]. Unfortunately, this type of cancer is typically asymptomatic in its early stages, making it challenging to detect early on. As the tumor grows, individuals may experience changes in vision, irritation, redness, or the sensation of a foreign body in the eye. Conjunctival melanoma has the potential to spread to other parts of the eye and nearby tissues, and in advanced stages, it can metastasize to distant organs [4–6].

Conjunctival melanoma is a rare form of cancer, and there is currently no evidence to support the idea that it shares the same risk factors as skin melanoma (such as exposure to ultraviolet radiation, fair skin, and a history of melanoma or other skin cancers). To diagnose conjunctival melanoma, a comprehensive eye examination is necessary, including the use of a slit lamp biomicroscope and imaging studies. Biopsy and histological examination are crucial for confirming the diagnosis and determining the extent of the disease. Staging can help guide treatment decisions, which may involve surgery, radiation therapy, and in some cases, immunotherapy or targeted therapy [7–9].

The current study presents two rare cases of amelanotic conjunctival malignant melanoma. This variant of melanoma exhibits no discernible pigmentation, thereby complicating the diagnostic process.

## Case presentation

### Case 1

**Fig. 1:**
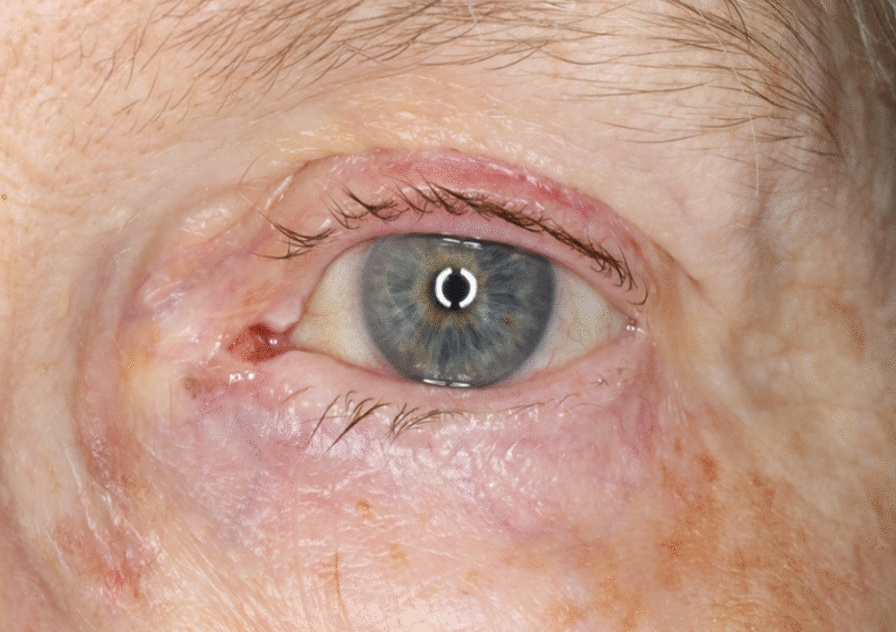
Photograph of the patient’s left eye showing redness in the upper eyelid

An 81-year-old Swedish-born woman visited the Ophthalmology Department at Skaraborg Hospital (Skövde) because of redness and a foreign body sensation in her left eye. During the examination, her vision was measured at 1.0 in the right eye and 0.6 in the left eye (using Snellen’s chart) while wearing her glasses. The slit lamp biomicroscope examination revealed that her upper eyelid was thicker and redder than the other eyelid, and she was diagnosed with blepharitis. The patient was treated with a combination of corticosteroid and antibiotic ointment and was scheduled for a follow-up appointment in 2–3 weeks. During the follow-up examination, the patient reported feeling better but still experiencing redness and a foreign body sensation in her left eye. See Fig. 1 for further details.

**Fig. 2:**
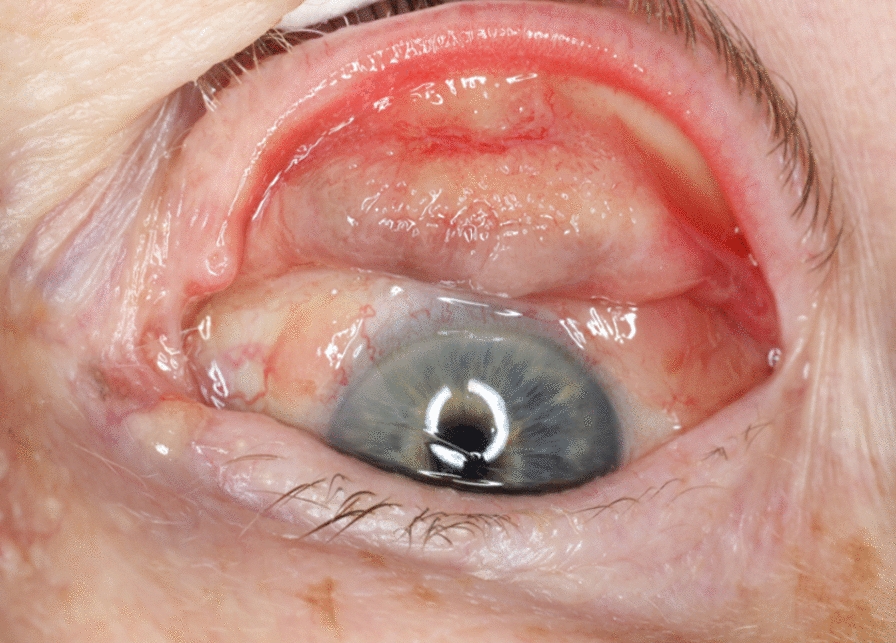
Image depicting the everted upper eyelid, revealing a palpebral conjunctiva tumor

A colleague sought consultation from the oculoplastic section (MA) as the patient showed no improvement. During the examination of the upper eyelid, a tumor-like growth was identified. Subsequently, three biopsies were taken from the eyelid under local anesthesia and immediately sent to the pathologist for analysis. It was assumed that the rest of the eye had already been examined. Please refer to Fig. 2 for a visual representation of the eyelid thickness caused by the tumor. The biopsies revealed a subepithelial growth of severely atypical melanocytes, consistent with amelanotic conjunctival malignant melanoma.

The Eye Department team at Sahlgrenska University Hospital in Gothenburg was contacted for the patient’s follow-up. The hospital’s pathologists were asked to confirm the diagnosis by reviewing the glasses. Additionally, magnetic resonance imaging (MRI) scans were ordered to assess any potential cancer spread to the orbit. In addition, computed tomography (CT) scans of the neck, abdomen, and thorax were ordered to detect any cancerous spread beyond the eye region.

**Fig. 3:**
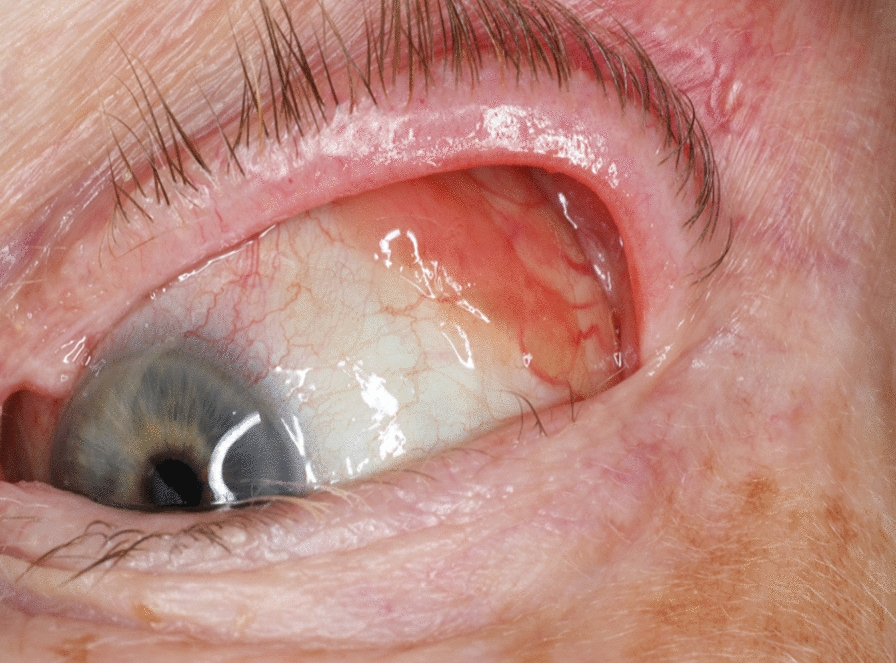
Figure showing conjunctival changes. The conjunctival lesions are in the upper temporal, and the unpigmented lesion can be observed

**Fig. 4:**
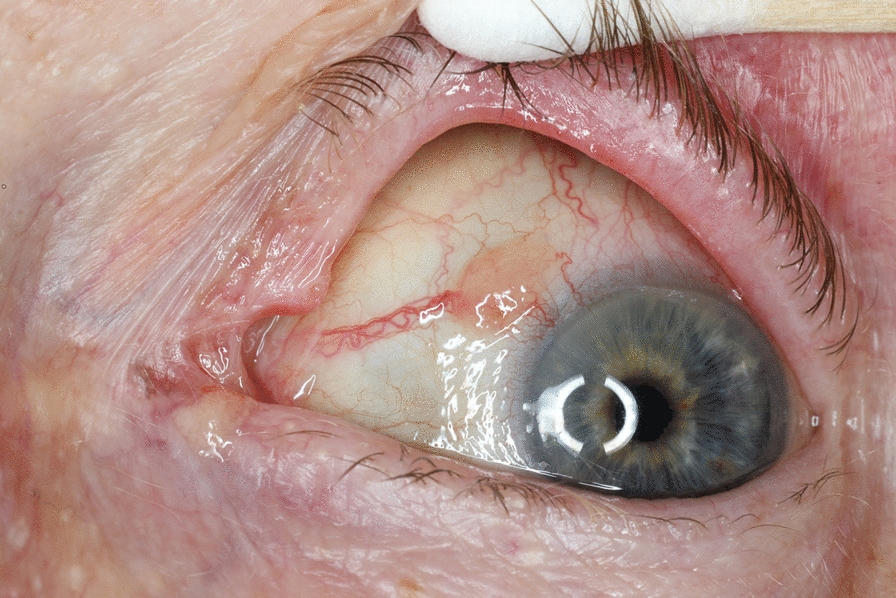
Figure showing conjunctival lesions located upper nasally; the unpigmented lesion can be observed

After 2 weeks, the patient was examined at the Eye Department of the Sahlgrenska University Hospital (KE). The visual acuity remained unchanged. However, the examination revealed the presence of slight, brown-pigmented lesions in the upper conjunctiva, both temporal and nasal. These lesions were not diagnosed in the two previous visits, as the focus was on the eyelid. The images of the affected area are in Figs. 3 and 4.

Fortunately, the rest of the eye was undamaged, and the fundus of the eye was normal. The right eye also showed no signs of cancer. However, the MRI orbit indicated the possibility of tumor metastasis to the lateral rectus muscle. On the contrary, the CT scans of the neck, abdomen, and thorax showed no signs of metastasis.

**Fig. 5:**
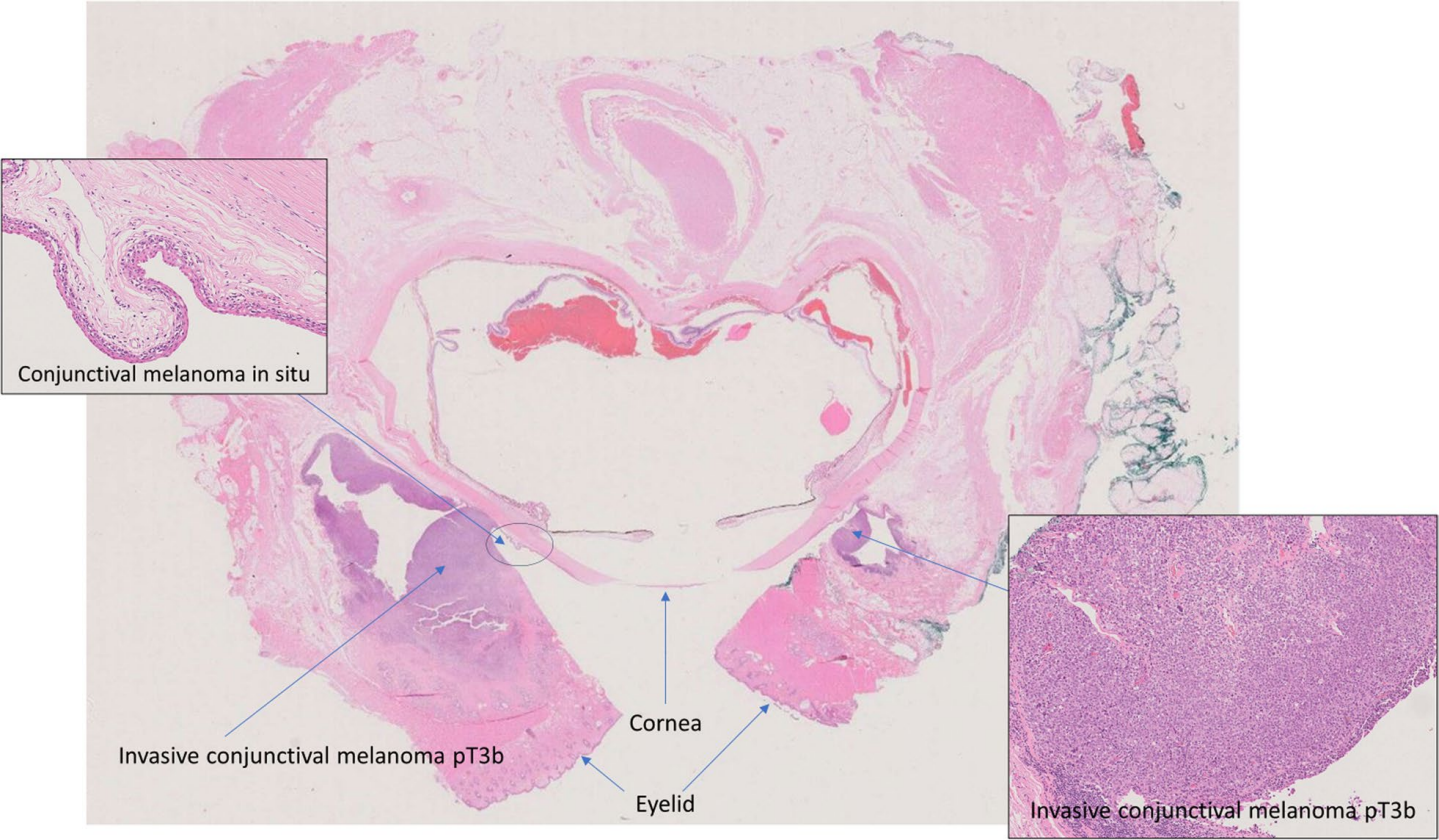
Figure showing histopathology of the eye after exenteration. It reveals conjunctival melanoma in situ in the eye and invasive conjunctival melanoma in the eyelid

**Fig. 6:**
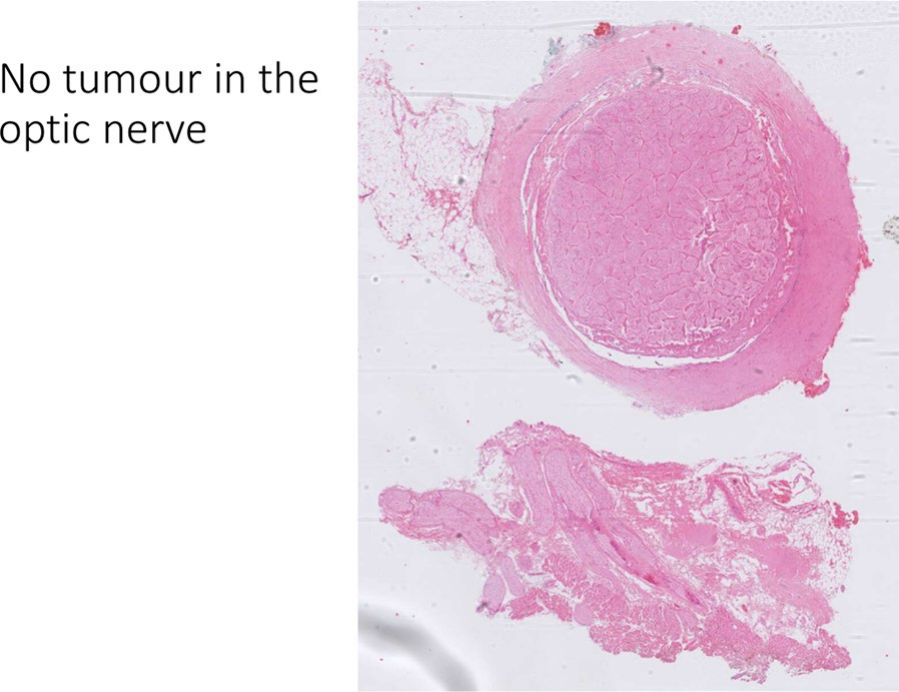
Figure displaying the histopathology of the eye after exenteration. The image depicts that no tumor invasion of the optic nerve was found

The patient’s fall was consulted with colleagues at the St Erik’s Eye Hospital, Stockholm. They recommended immunotherapy for the patient. A multidisciplinary meeting was held at Sahlgrenska University Hospital to discuss the case. The meeting determined that orbital exenteration was the best approach because a procedure that saved the eye would not completely remove the tumor. Additionally, evidence for using immunotherapy for conjunctival malignant melanoma was limited. As a result, the patient had orbital exenteration 2 weeks after the meeting. The eye, muscles, and orbital contents were sent for histological analysis. The biopsy from the palpebral conjunctiva revealed a small number of abnormal melanocytes within the epithelium, as well as a highly variable population of epithelioid melanocytes underneath the epithelium (S-100+, SOX10+, Melan A+, Ki67+). The specimen from the surgical removal of the eye showed extensive spread of malignant melanoma in the eyelid and bulbar conjunctiva, measuring 13 mm wide and 7 mm deep, with a 4 mm margin removed. Please refer to Fig. 5 for more details. Fortunately, there was no tumor growth within the optic nerve. See Fig. 6.

The findings confirmed that the patient had an amelanotic conjunctival malignant melanoma. The surgery was radical, and it was determined that the patient did not require any additional therapy; 9 months later, the patient is doing well, with no signs of metastasis identified, and is using a prosthesis. The patient is satisfied with the cosmetic results of the intervention.

### Case 2

A 50-year-old white Swedish woman had a lesion on her left eye, which caused redness and a feeling of having something in her eye. The lesion had been present for about 2–3 months when she visited the primary healthcare unit. The general physician suspected pinguecula and referred the patient to the Department of Ophthalmology at the communal hospital of Borås.

During a clinical ophthalmological examination, the slit lamp biomicroscopy revealed a conjunctival lesion of approximately 2 mm × 4.8 mm on the temporal bulbar conjunctiva of the left eye. The lesion did not extend over the cornea and was freely movable against the underlying sclera at that point of examination. The patient was treated with antibiotic ointment and lubricants. However, the clinical appearance of the lesion was not typical for a pinguecula. As a result, the patient was referred to the Department of Ophthalmology at Sahlgrenska University Hospital in Gothenburg for further evaluation.

**Fig. 7:**
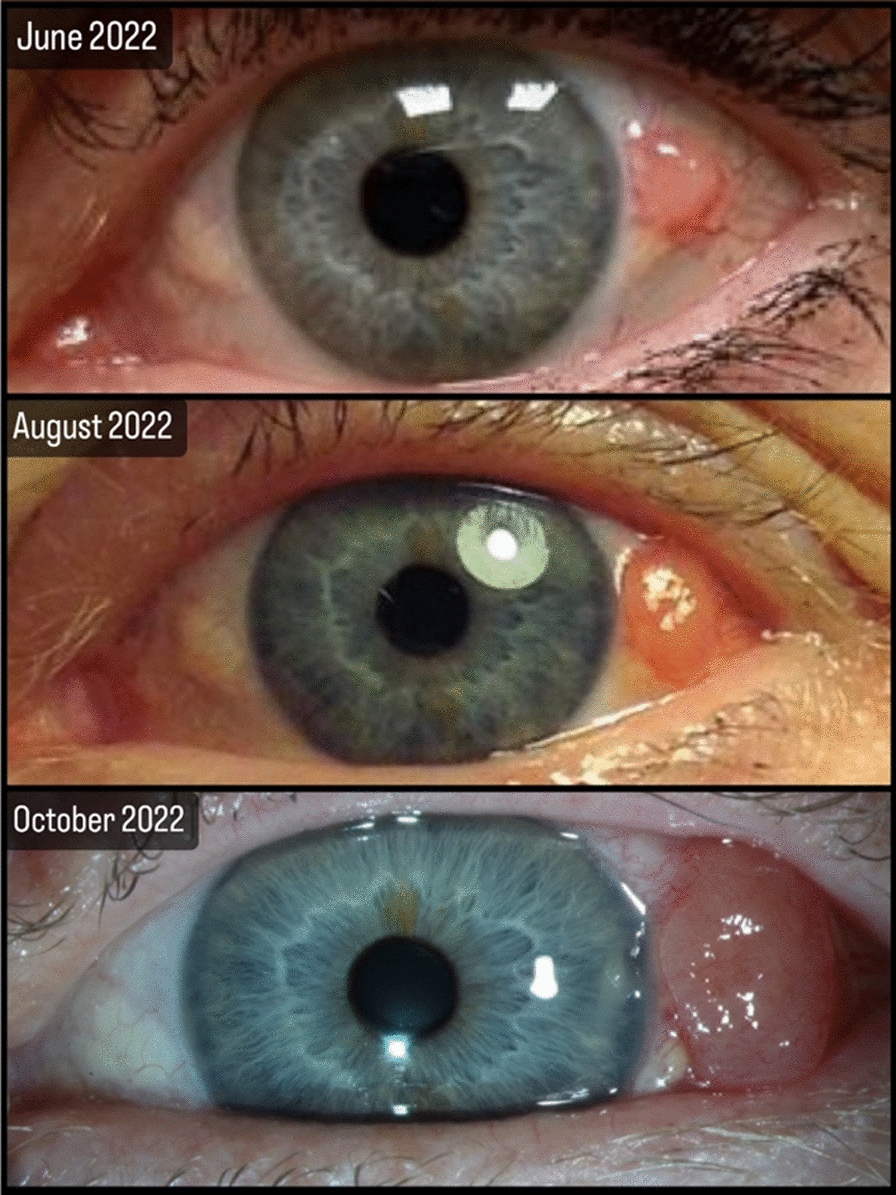
Figure showcasing the clinical presentation of the tumor in case 2. It is observed that the tumor grew 2–3 times its size in just 4 months

During the clinical ophthalmological examination, a lesion of approximately 4 mm × 5 mm was found on the temporal bulbar conjunctiva near the limbus. The lesion was still mobile against the underlying sclera but adherent against tenon. Since the patient’s initial visit to the primary healthcare unit, the tumor had grown (see Fig. 7). The patient showed no clinical signs of lymphadenopathy during palpation of the regional lymph nodes, and visual acuity was 1.0 (measured using Snellen’s chart) in both eyes.

On the basis of these clinical signs, there was a strong suspicion of malignancy, specifically conjunctival intraepithelial neoplasia (CIN). Therefore, it was highly recommended that the patient undergo further surgery. The surgery was performed under local anesthesia using the no-touch technique [10]. The tumor was removed with a 3.5 mm free margin, including the margin over the corneal epithelium, which was removed by localized alcohol corneal epitheliectomy. The specimen was sent to St. Erik Eye Hospital in Stockholm for histopathological analysis. Before the surgery, no MRI or CT scans were conducted.

**Fig. 8:**
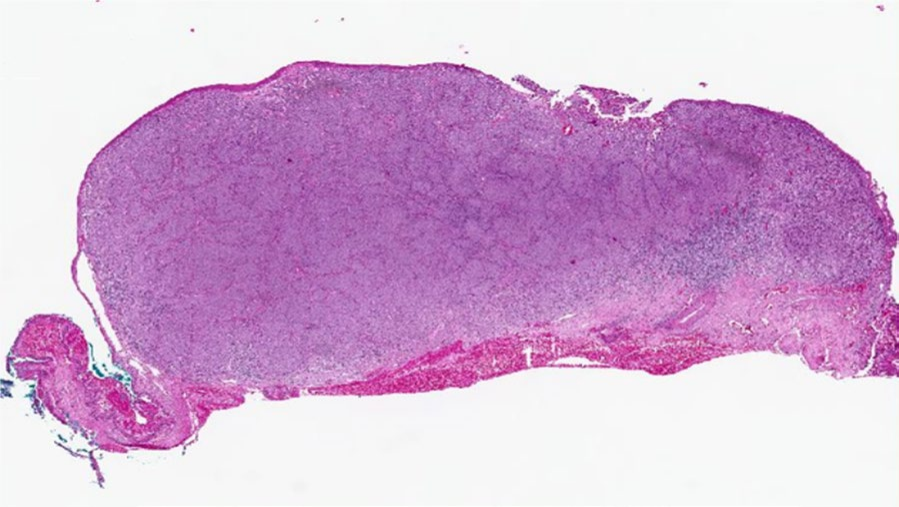
Figure demonstrating the histopathology of the conjunctival tumor after excision. It is an image showing an ulcerated nodular conjunctival melanoma that is 3.2 mm deep, H&E, original magnification 100×

The histopathological analysis revealed the presence of an amelanotic nodular conjunctival melanoma measuring 6.8 mm in diameter and 3.2 mm deep. The surface showed focal ulceration, with nine mitoses per ten high-power fields, placing it at stage 2B. The tumor cells displayed tightly packed, highly atypical features with a spindled morphology and prominent nucleoli. The deep margin was very narrow, at most 0.05 mm. Complete removal of the tumor at the temporal or medial end of the specimen could not be guaranteed. However, the cancer was removed with a margin of at least 0.6 mm cranially and 1 mm caudally. Please refer to Fig. 8 for further details.

Following the analysis, the patient was advised to undergo adjuvant postoperative radiotherapy. About 2.5 months after the initial surgery, the patient received brachytherapy with a Ruthenium applicator on the conjunctiva of the left eye at St. Erik Eye University Hospital in Stockholm. Approximately 1 month after brachytherapy, the patient had additional therapy with mitomycin C 0.04%, which was tolerated well with no reported adverse effects.

An initial CT scan of the thorax, neck, and abdomen showed no metastasis. A follow-up MRI of the brain and orbit 3 months after the surgery also revealed no signs of metastasis or tumor recurrence. However, the patient did have a slightly palpable nodule on one of the left supraclavicular lymph nodes, which was later determined to be benign after a sentinel node biopsy. About 4 months after receiving mitomycin treatment, a control biopsy of the left eye’s conjunctiva was performed, and none of the four conjunctival biopsies showed any sign of tumor recurrence.

**Fig. 9:**
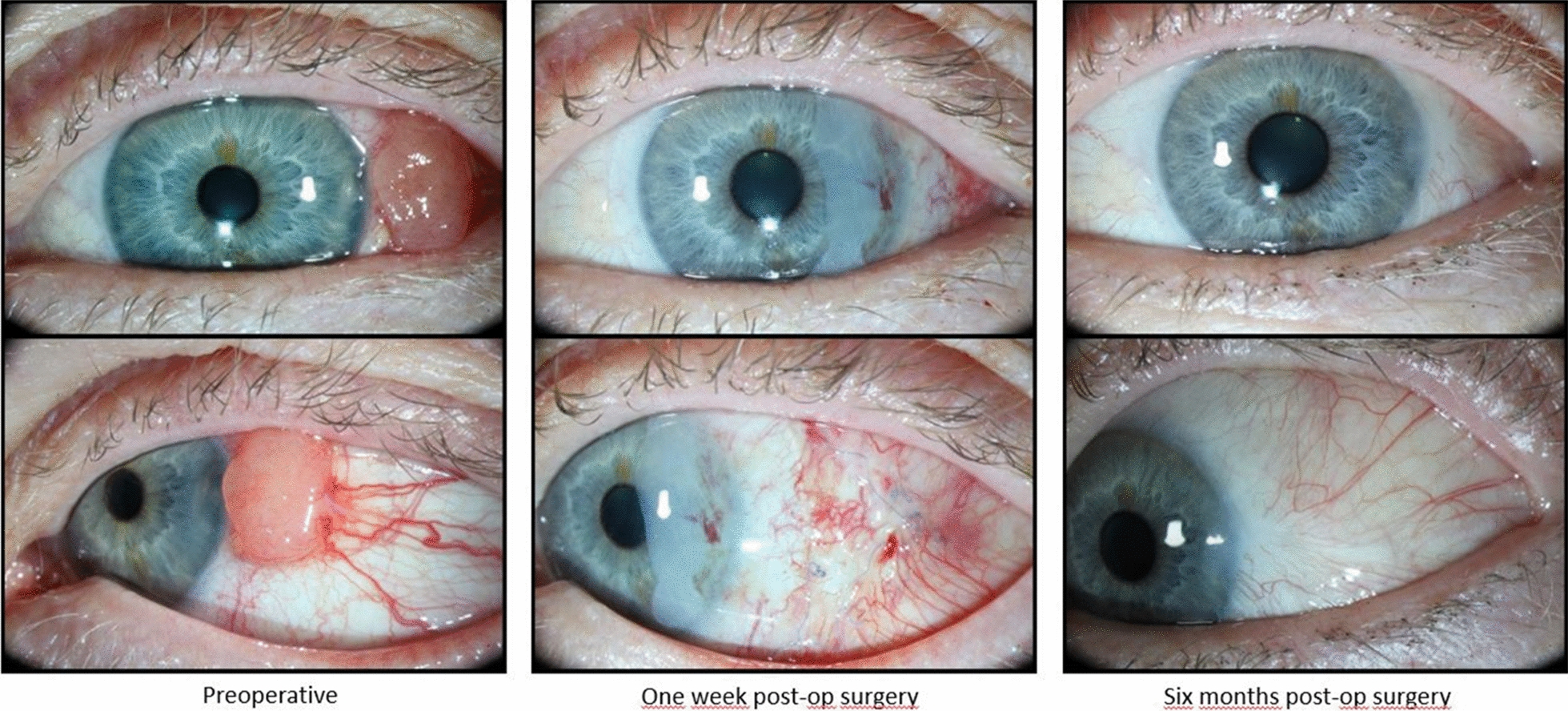
Figure highlighting the clinical presentation of case 2. It is shown preoperatively on the left, 1 week after surgery in the middle, and 6 months after surgery on the right

The patient has been undergoing a complete ophthalmological examination every 3 months, including lymph node palpation. The visual acuity of the left eye has been fully preserved, and the patient has tolerated the additional treatments (brachytherapy and mitomycin) well. After 1 year and 7 months, the patient is doing well and is satisfied with the results. No signs of metastasis were identified, and the left eye has a visual acuity of 1.0 on Snellen’s chart. The patient will have follow-up appointments every 6 months for 5 years. If no signs of tumors are present during this time, the patient will have annual follow-ups for the rest of her life. For more details, please refer to Fig. 9.

## Discussion

This article reports on two cases of women who had a rare and uncommon form of cancer: unilateral amelanotic malign melanoma in the conjunctiva. A search in PubMed (https://pubmed.ncbi.nlm.nih.gov) revealed only five published articles on this topic, with the oldest dating back to 1990. Of these, only one article included four cases, while the other articles were based on individual patient reports. This highlights the rarity of amelanotic conjunctival melanoma [11–15].

Cutaneous amelanotic melanomas, which are melanomas of the skin that lack pigmentation, account for approximately 2–8% of all melanomas. The free surgical margin is conjunctival melanoma’s most critical favorable prognostic factor. Factors such as deep invasion, presence of pagetoid melanoma in situ, amelanotic PAM, intravasal growth, or a high mitotic index are associated with worse outcomes [16].

The lack of melanin production in the body is linked to less specialized and more aggressive tumors. It is difficult to diagnose these tumors, which can lead to delays in treatment, especially for skin tumors. Because there have been few cases, we need to understand better the clinical and molecular features of these lesions, including their impact on mortality, the spread of cancer, and the effectiveness of treatments. We need more research to understand how these tumors develop and to create better ways to diagnose and treat this type of skin cancer.

Detecting amelanotic lesions in the conjunctiva can be quite challenging, as these lesions are not visible. Patients might not be aware of their presence, and doctors may be unable to identify them during routine examinations. There is a risk of misidentifying these lesions as benign conjunctival changes, such as pingueculas or harmless eyelid lesions.

In the first case described, biopsies were taken, and sebaceous gland carcinoma or lymphoma were the two possible diagnoses considered. Malignant melanoma was not suspected, and it was not included in the clinical differential diagnosis. The initial diagnosis was pinguecula in the second case, but conjunctival intraepithelial neoplasia (CIN) was suspected during the lesion removal. The no-touch technique was thus used. In both cases, malignant melanoma was not suspected.

Risk factors for cutaneous malignant melanomas have been extensively described in the literature. However, it is difficult to identify specific risk factors due to the rarity of conjunctival malign melanomas and even more for amelanotic lesions. Pacheco et al. followed 629 patients suffering from conjunctival melanoma in the lapse of 5 years [3]. Only 46 patients were suffering from amelanotic lesions, which shows how rare this presentation is. The most extensive study on conjunctival malign melanomas published, based on a Scandinavian population in Denmark, was conducted by Larsen A.C. in 2016 [1]. The Danish cohort study followed 139 patients diagnosed over 52 years (1960–2012). The median age of the patients was 67 years, ranging from 14 to 100 years. According to the study, only 19% of the cases (*n* = 20) were nonpigmented, with no gender prevalence. The study suggested that conjunctival and skin melanoma might share common pathogenic pathways, indicating potentially similar risk factors for both. However, no similarities were found with choroidal melanomas. The study also suggested that immunotherapy, which has been effective in treating cutaneous melanomas, might be a viable treatment option for conjunctival melanomas. The present study included two women of different ages who denied any solar exposure. The current literature debates the significance of solar exposure in developing conjunctival melanomas.

In the first case, the patient underwent orbital exenteration as a treatment. However, systemic immunotherapy could be an alternative treatment for disseminated or multifocal disease, especially in skin melanomas. There is limited evidence of immunotherapy for conjunctival melanomas, but some case reports exist. This therapy works by reducing the activity of T cells, and the two most used inhibitors are PD-1 and CTLA-4. Common side effects of these medications include immune-mediated pneumonitis, colitis, hepatitis, nephritis, skin rash, and thyroid dysfunction.

The oncologists at Sahlgrenska Hospital had limited evidence of the effectiveness of systemic immunotherapy. They recommended using it as an adjuvant treatment only if the tumor could not be radically operated.

In the second case, complete removal of the tumor toward the temporal or medial end of the specimen was not possible. Therefore, adjuvant therapy was recommended. Brachytherapy was used initially, and after a month, local chemotherapy (mitomycin C eye drops) was also administered [17]. The patient’s eye retained a visual acuity of 1.0 (according to Snellen’s chart). However, further evidence is needed to determine the effectiveness of immunotherapy in treating amelanotic conjunctival malign melanomas.

In the second case, the central mass was removed, and the sclera was treated with alcohol application. Cryotherapy was performed from the underside of the conjunctival resection ends, followed by reconstruction using an amniotic membrane allogeneic transplant. A no-touch method was utilized, and direct tumor manipulation was avoided to prevent tumor cells from spreading to a new area. The no-touch technique was used, as CIN was suspected. This well-planned initial surgical management technique reduces the chance of tumor recurrence for conjunctival malignancy. Despite the unverified diagnosis at the time of surgery, the no-touch technique was performed due to a high suspicion of conjunctival malignancy [10].

Ocular melanoma is a condition that can present itself in two subtypes: conjunctival melanoma and uveal melanoma. These subtypes have different genetic pathways that are responsible for their progression. Conjunctival melanoma is closely associated with malignant melanoma that arises in sun-exposed skin and is characterized by the activation of the MAPK (RAS/RAF/MEK/ERK) or PI3K/AKT/mTOR pathways. Furthermore, the other subtype is unrelated to ultraviolet (UV) exposure and has similarities to lentiginous melanoma that arises in mucosal surfaces. This subtype is characterized by mutations in TERT and ATRX. Thus, understanding these genetic pathways is critical to diagnosing and managing ocular melanoma [18]. Regrettably, the two cases under consideration did not undergo molecular analysis. Nevertheless, the diagnosis and clinical management remained unchanged. Histopathology and surgical excision still stand as the gold standard [19].

## Conclusions

This report emphasizes the significance of considering amelanotic conjunctival malignant melanoma when changes in the conjunctiva and eyelids are noticed. The authors’ findings serve as a reminder to clinicians to be vigilant in their evaluations and to promptly initiate treatment to minimize the risk of metastasis and improve patient outcomes.

## Data Availability

All data generated or analyzed during this study are included in this published article.

## Abbreviations

MRI: Magnetic resonance imaging
CT: Computed tomography
CIN: Conjunctival intraepithelial neoplasia
PAM: Primary acquired melanosis
HPF: High-power fields

## References

[1] Larsen AC. Conjunctival malignant melanoma in Denmark: epidemiology, treatment and prognosis with special emphasis on tumorigenesis and genetic profile. Acta Ophthalmol. 2016;94:1–27. 27192168 10.1111/aos.13100

[2] Seregard S, Kock E. Conjunctival malignant melanoma in Sweden 1969–91. Acta Ophthalmol (Copenh). 1992;70(3):289–96. 1636385 10.1111/j.1755-3768.1992.tb08566.x

[3] Pacheco RR, Yaghy A, Dalvin LA, Vaidya S, Perez AL, Lally SE, *et al*. Conjunctival melanoma: outcomes based on tumour origin in 629 patients at a single ocular oncology centre. Eye. 2022;36(3):603–11. 33772241 10.1038/s41433-021-01508-y PMC8873502

[4] El Ouazzani H, Janah H, El Machichi SA, Achachi L, Fihry MT, El Ftouh M. Pleuropulmonary metastasis revealing a malignant melanoma of the conjunctiva in a young subject. Pan Afr Med J. 2016;23:98. 27231507 10.11604/pamj.2016.23.98.7246 PMC4867732

[5] Mizuuchi H, Suda K, Kitahara H, Shimamatsu S, Kohno M, Okamoto T, *et al*. Solitary pulmonary metastasis from malignant melanoma of the bulbar conjunctiva presenting as a pulmonary ground glass nodule: report of a case. Thorac Cancer. 2015;6(1):97–100. 26273342 10.1111/1759-7714.12124 PMC4448462

[6] Manidakis N, Polyzois I, Tsialogiannis E, Marples M, Boon A, Tsiridis E. Metastatic malignant melanoma of the conjunctiva: a case report. Cases J. 2009;2(1):125. 19193228 10.1186/1757-1626-2-125 PMC2642788

[7] Chalasani R, Giblin M, Conway RM. Role of topical chemotherapy for primary acquired melanosis and malignant melanoma of the conjunctiva and cornea: review of the evidence and recommendations for treatment. Clin Exp Ophthalmol. 2006;34(7):708–14. 16970772 10.1111/j.1442-9071.2006.01356.x

[8] Zografos L, Uffer S, Bercher L, Gailloud C. Combined surgery, cryocoagulation and radiotherapy for treatment of melanoma of the conjunctiva. Klin Monbl Augenheilkd. 1994;204(5):385–90. 8051878 10.1055/s-2008-1035564

[9] Scholz SL, Hérault J, Stang A, Griewank KG, Meller D, Thariat J, *et al*. Proton radiotherapy in advanced malignant melanoma of the conjunctiva. Graefes Arch Clin Exp Ophthalmol. 2019;257(6):1309–18. 30919076 10.1007/s00417-019-04286-2

[10] Shields JA, Shields CL, De Potter P. Surgical management of conjunctival tumors: the 1994 Lynn B. McMahan Lecture. Arch Ophthalmol. 1997;115(6):808–15. 9194740 10.1001/archopht.1997.01100150810025

[11] Jordan DR, Mamalis N, White GL Jr, Hansen SO, Anderson RL. Amelanotic malignant melanoma of the conjunctiva with local metastasis to the eyelid. Can J Ophthalmol. 1990;25(1):34–7. 2328436

[12] Paridaens AD, McCartney AC, Hungerford JL. Multifocal amelanotic conjunctival melanoma and acquired melanosis sine pigmento. Br J Ophthalmol. 1992;76(3):163–5. 1540561 10.1136/bjo.76.3.163 PMC504196

[13] Bongiorno MR, Lodato G, Affronti A, Aragona F, Aricò M. Amelanotic conjunctival melanoma. Cutis. 2006;77(6):377–81. 16838771

[14] Barros Jde N, Motono M, Costa FD, Cunha MC, Chojniak MM. Amelanotic corneally displaced malignant conjunctival melanoma: a case report evaluated with impression cytology. Arq Bras Oftalmol. 2014;77(1):57–9. 25076376 10.5935/0004-2749.20140015

[15] Kovacević D, Lukanović-Primc K, Markusić V, Babić MB, Ledić D. Conjunctival amelanotic melanoma–a case report. Coll Antropol. 2011;35(Suppl 2):295–7. 22220456

[16] Layton C, Glasson W. Clinical aspects of conjunctival melanoma. Clin Exp Ophthalmol. 2002;30(2):72–9. 11886408 10.1046/j.1442-6404.2002.00490.x

[17] Shields CL, Shields JA. Tumors of the conjunctiva and cornea. Indian J Ophthalmol. 2019;67(12):1930–48. 31755426 10.4103/ijo.IJO_2040_19 PMC6896532

[18] Brouwer NJ, Verdijk RM, Heegaard S, Marinkovic M, Esmaeli B, Jager MJ. Conjunctival melanoma: new insights in tumour genetics and immunology, leading to new therapeutic options. Prog Retin Eye Res. 2022;86:100971. 34015548 10.1016/j.preteyeres.2021.100971

[19] Wong JR, Nanji AA, Galor A, Karp CL. Management of conjunctival malignant melanoma: a review and update. Expert Rev Ophthalmol. 2014;9(3):185–204. 25580155 10.1586/17469899.2014.921119 PMC4285629

